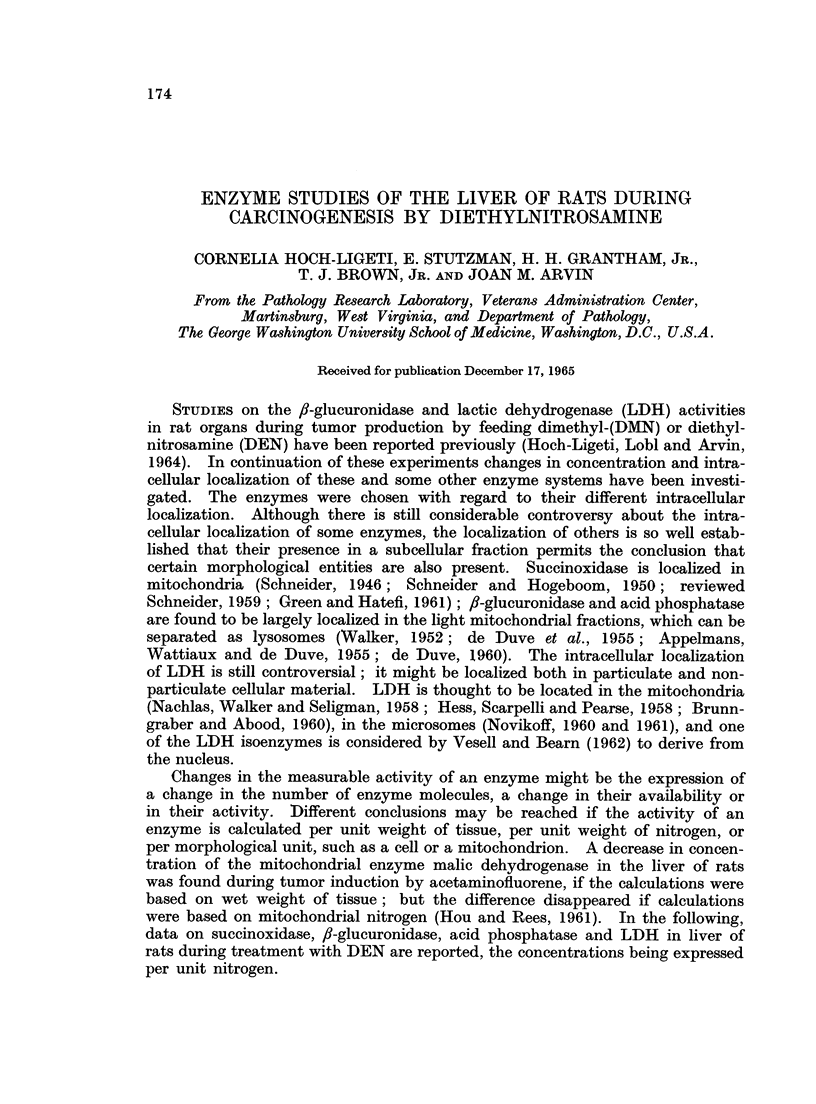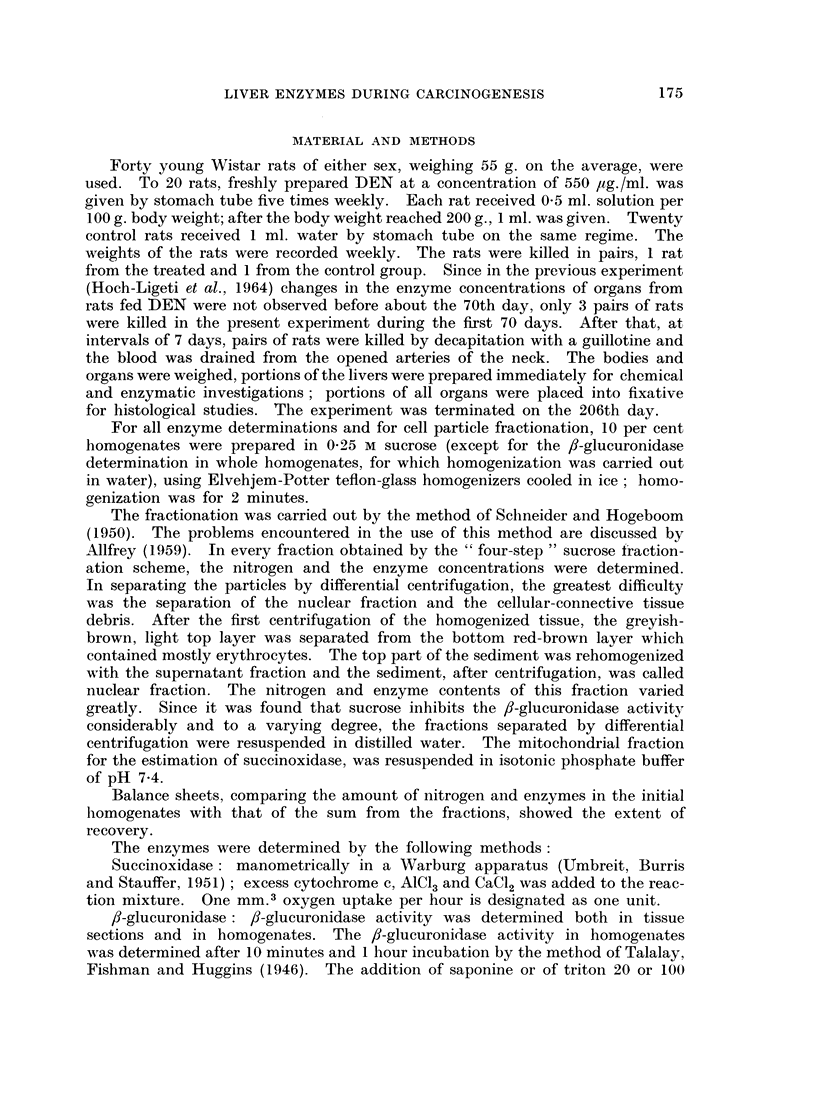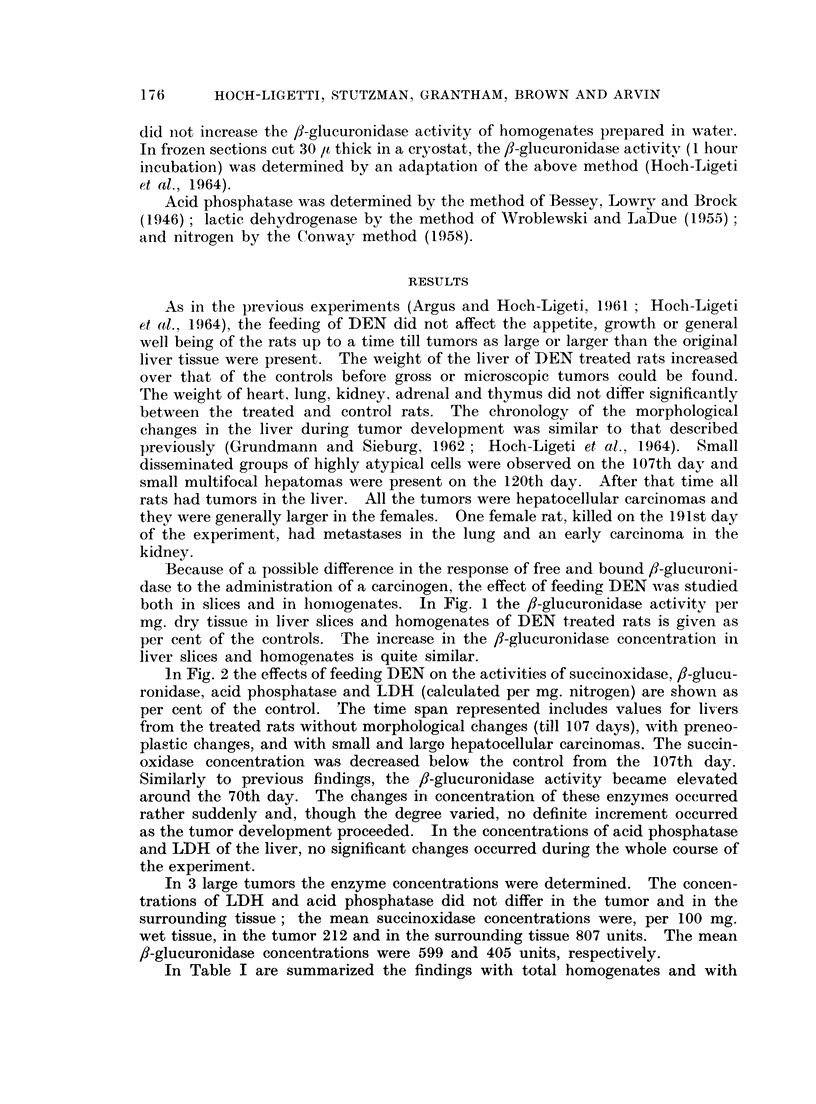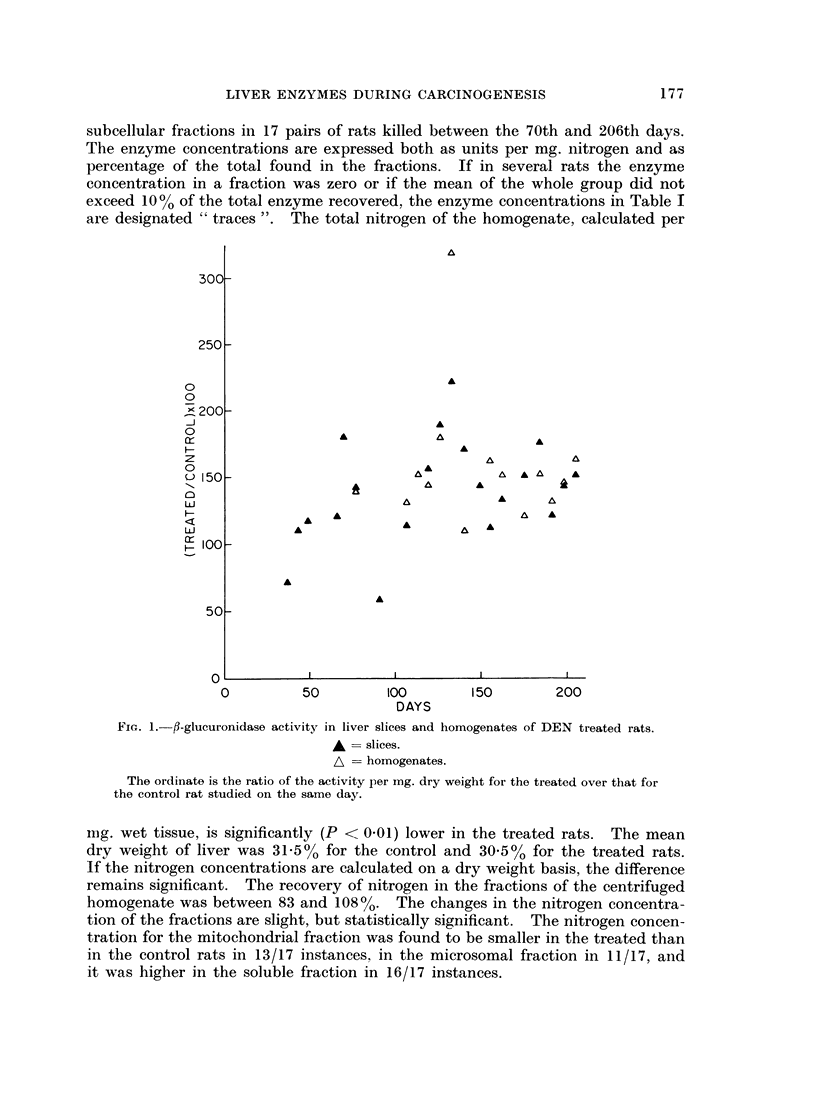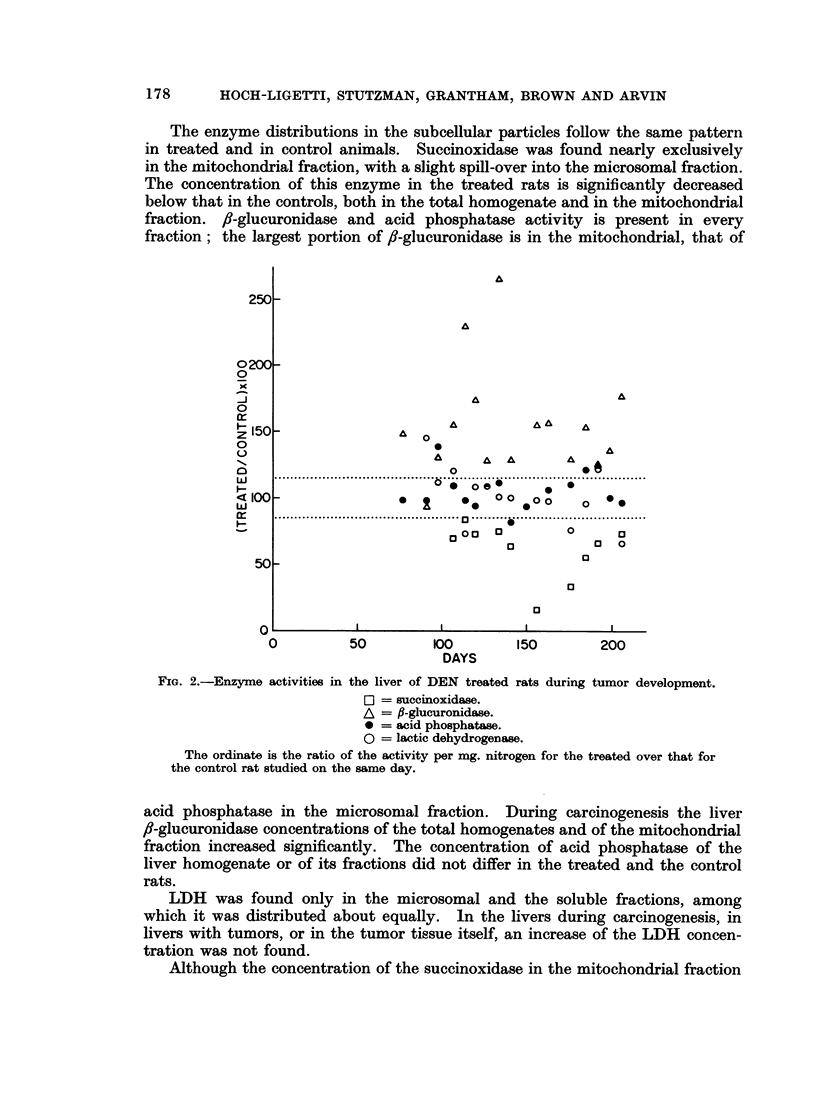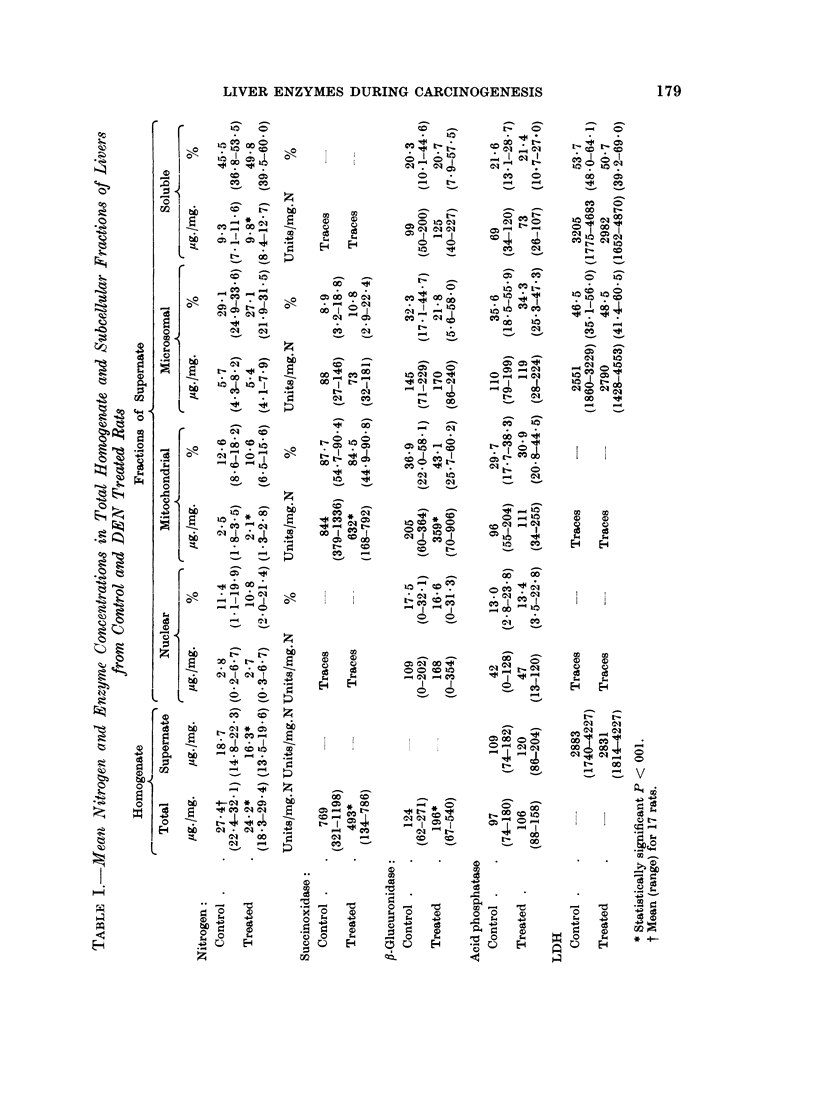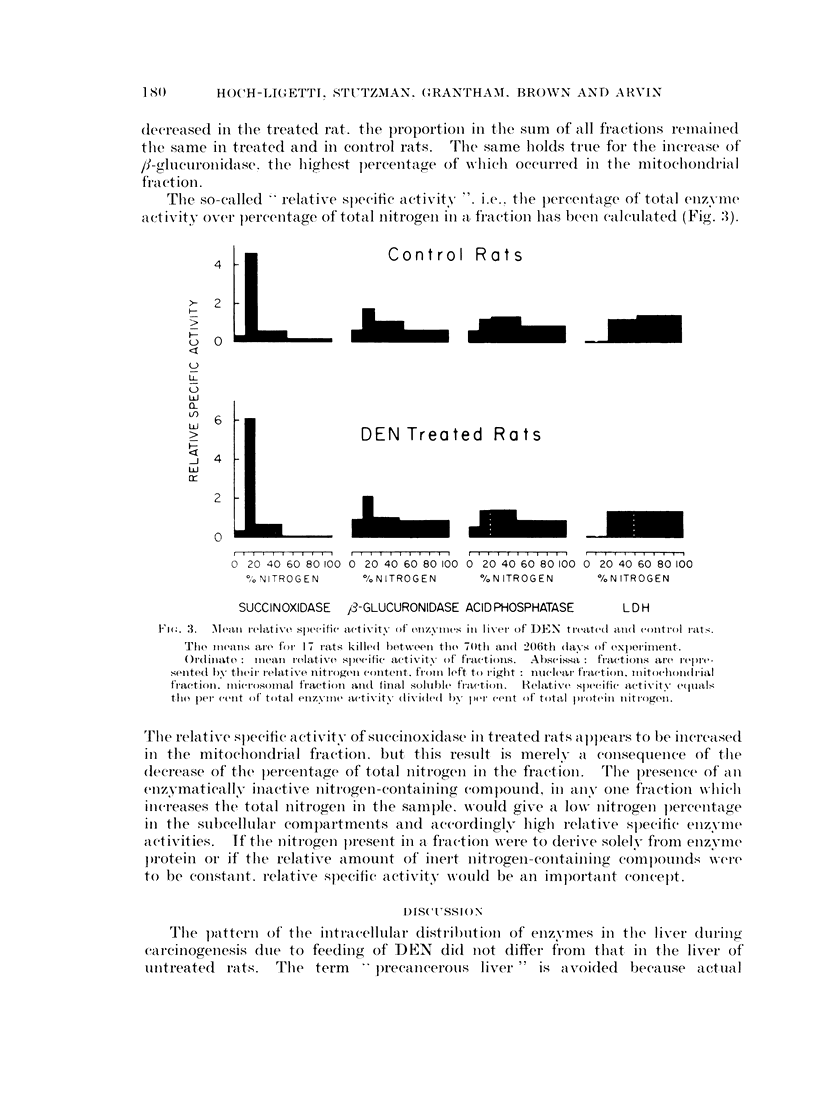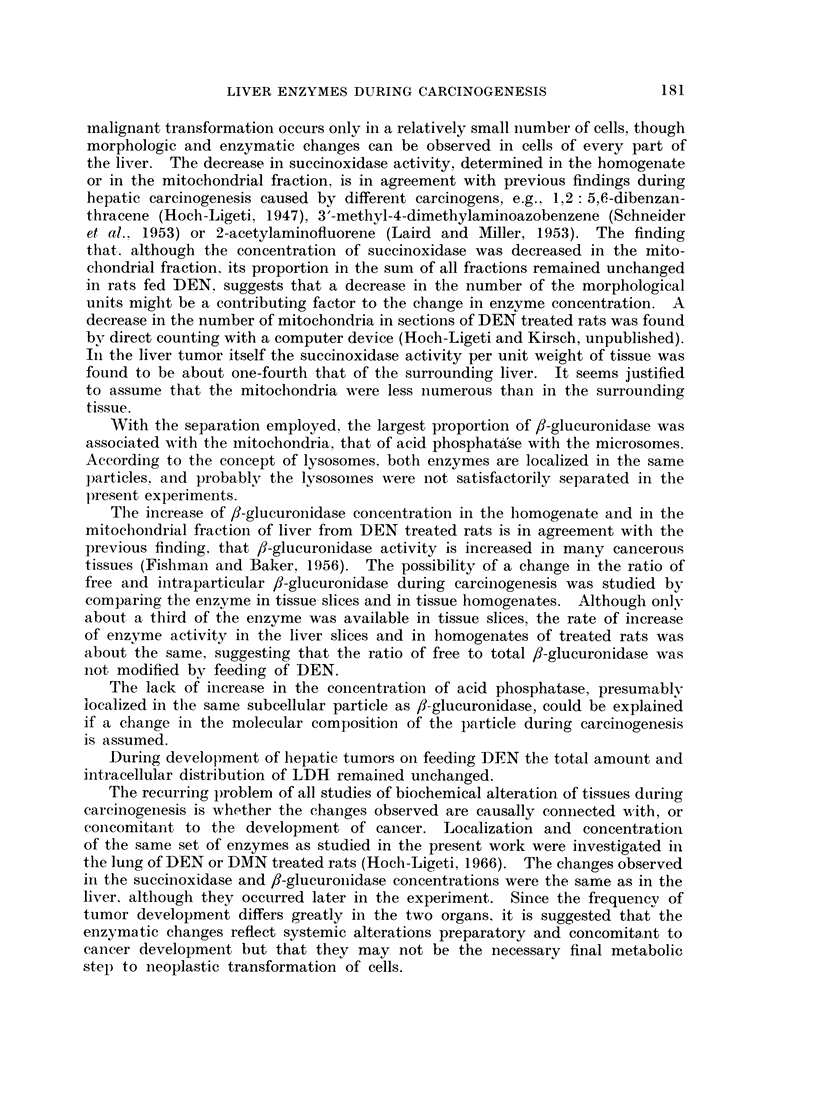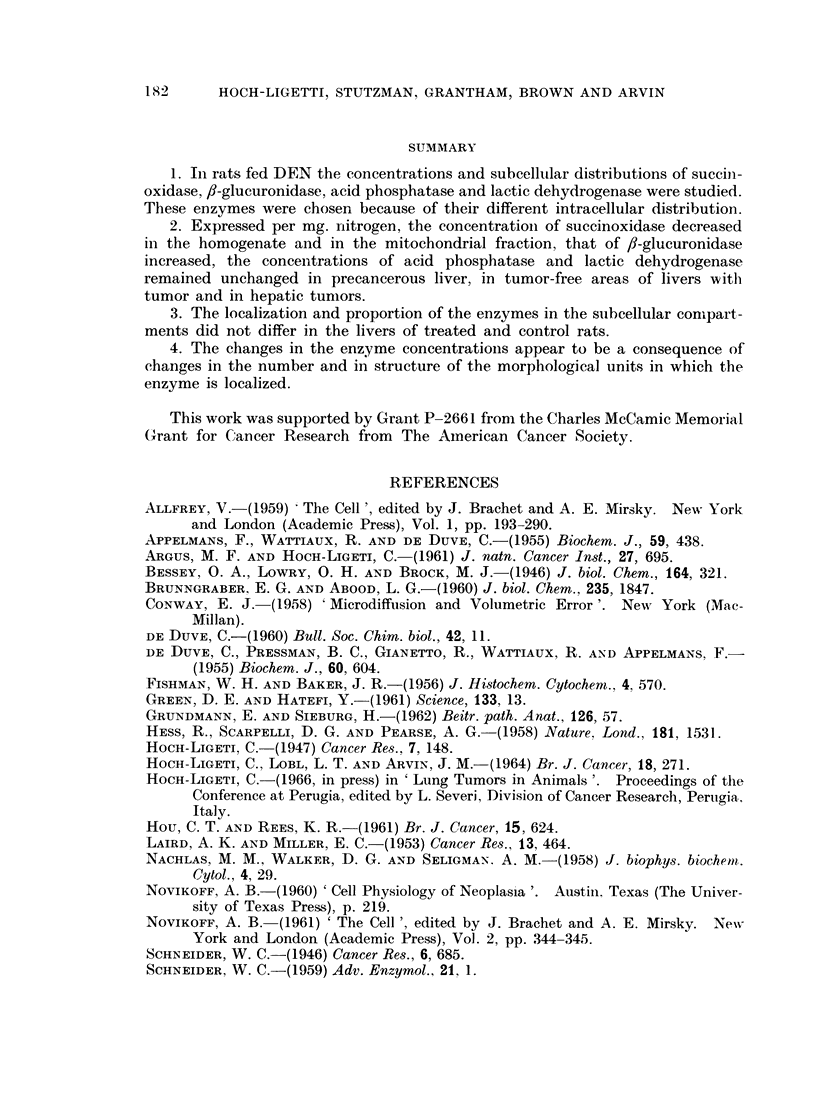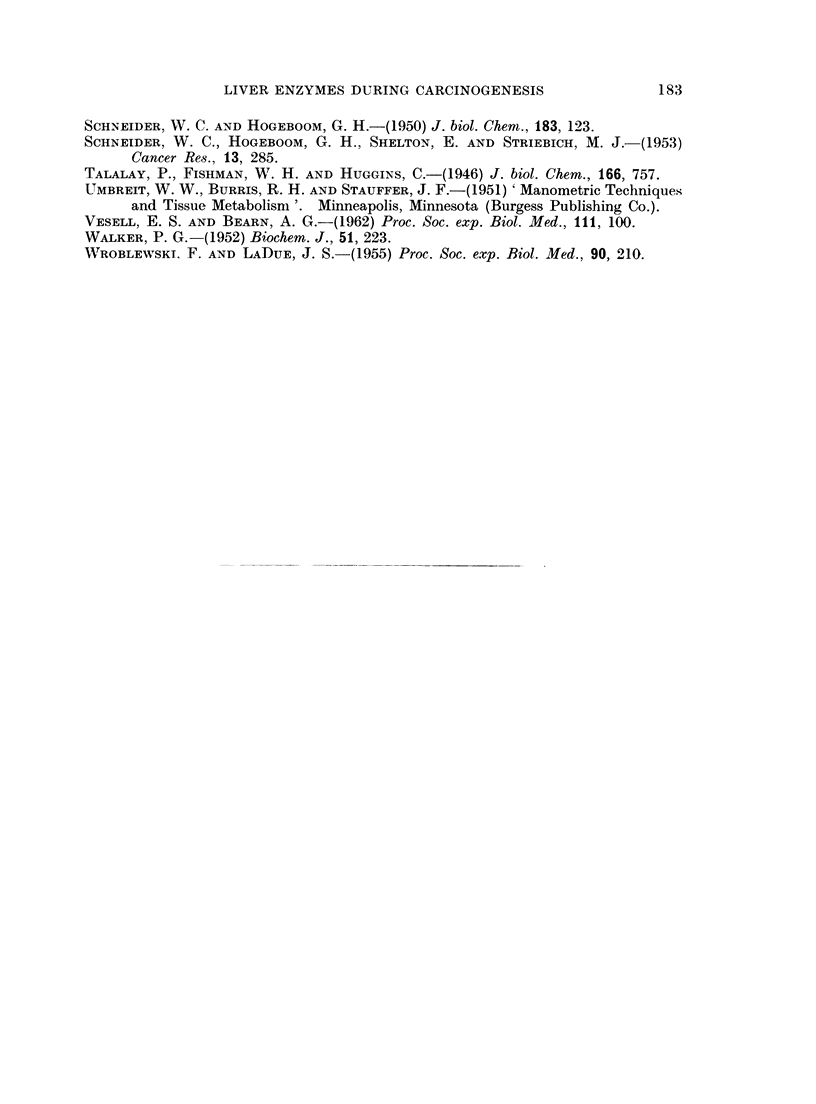# Enzyme studies of the liver of rats during carcinogenesis by diethylnitrosamine.

**DOI:** 10.1038/bjc.1966.20

**Published:** 1966-03

**Authors:** C. Hoch-Ligeti, E. Stutzman, H. H. Grantham, T. J. Brown, J. M. Arvin


### ENZYME STUDIES OF THE LIVER OF RATS DURING CARCINOGENESIS BY DIETHYLNITROSAMINE

CORNELIA HOCH-LIGETI, E. STUTZMAN, H. H. GRANTHAM, JR., T. J. BROWN, JR. AND JOAN M. ARVIN

*From the Pathology Research Laboratory, Veterans Administration Center, Martinsburg, West Virginia, and Department of Pathology, The George Washington University School of Medicine, Washington, D.C., U.S.A.*

Received for publication December 17, 1965

STUDIES on the β-glucuronidase and lactic dehydrogenase (LDH) activities in rat organs during tumor production by feeding dimethyl-(DMN) or diethylnitrosamine (DEN) have been reported previously (Hoch-Ligeti, Lobl and Arvin, 1964). In continuation of these experiments changes in concentration and intracellular localization of these and some other enzyme systems have been investigated. The enzymes were chosen with regard to their different intracellular localization. Although there is still considerable controversy about the intracellular localization of some enzymes, the localization of others is so well established that their presence in a subcellular fraction permits the conclusion that certain morphological entities are also present. Succinoxidase is localized in mitochondria (Schneider, 1946; Schneider and Hogeboom, 1950; reviewed Schneider, 1959; Green and Hatefi, 1961); β-glucuronidase and acid phosphatase are found to be largely localized in the light mitochondrial fractions, which can be separated as lysosomes (Walker, 1952; de Duve *et al.*, 1955; Appelmans, Wattiaux and de Duve, 1955; de Duve, 1960). The intracellular localization of LDH is still controversial; it might be localized both in particulate and non-particulate cellular material. LDH is thought to be located in the mitochondria (Nachlas, Walker and Seligman, 1958; Hess, Scarpelli and Pearse, 1958; Brunngraber and Abood, 1960), in the microsomes (Novikoff, 1960 and 1961), and one of the LDH isoenzymes is considered by Vesell and Bearn (1962) to derive from the nucleus.

Changes in the measurable activity of an enzyme might be the expression of a change in the number of enzyme molecules, a change in their availability or in their activity. Different conclusions may be reached if the activity of an enzyme is calculated per unit weight of tissue, per unit weight of nitrogen, or per morphological unit, such as a cell or a mitochondrion. A decrease in concentration of the mitochondrial enzyme malic dehydrogenase in the liver of rats was found during tumor induction by acetaminofluorene, if the calculations were based on wet weight of tissue; but the difference disappeared if calculations were based on mitochondrial nitrogen (Hou and Rees, 1961). In the following, data on succinoxidase, β-glucuronidase, acid phosphatase and LDH in liver of rats during treatment with DEN are reported, the concentrations being expressed per unit nitrogen.

### MATERIAL AND METHODS

Forty young Wistar rats of either sex, weighing 55 g. on the average, were used. To 20 rats, freshly prepared DEN at a concentration of 550 $\mu$g./ml. was given by stomach tube five times weekly. Each rat received 0·5 ml. solution per 100 g. body weight; after the body weight reached 200 g., 1 ml. was given. Twenty control rats received 1 ml. water by stomach tube on the same regime. The weights of the rats were recorded weekly. The rats were killed in pairs, 1 rat from the treated and 1 from the control group. Since in the previous experiment (Hoch-Ligeti *et al.*, 1964) changes in the enzyme concentrations of organs from rats fed DEN were not observed before about the 70th day, only 3 pairs of rats were killed in the present experiment during the first 70 days. After that, at intervals of 7 days, pairs of rats were killed by decapitation with a guillotine and the blood was drained from the opened arteries of the neck. The bodies and organs were weighed, portions of the livers were prepared immediately for chemical and enzymatic investigations ; portions of all organs were placed into fixative for histological studies. The experiment was terminated on the 206th day.

For all enzyme determinations and for cell particle fractionation, 10 per cent homogenates were prepared in 0·25 M sucrose (except for the $\beta$-glucuronidase determination in whole homogenates, for which homogenization was carried out in water), using Elvehjem-Potter teflon-glass homogenizers cooled in ice ; homogenization was for 2 minutes.

The fractionation was carried out by the method of Schneider and Hogeboom (1950). The problems encountered in the use of this method are discussed by Allfrey (1959). In every fraction obtained by the " four-step " sucrose fractionation scheme, the nitrogen and the enzyme concentrations were determined. In separating the particles by differential centrifugation, the greatest difficulty was the separation of the nuclear fraction and the cellular-connective tissue debris. After the first centrifugation of the homogenized tissue, the greyish-brown, light top layer was separated from the bottom red-brown layer which contained mostly erythrocytes. The top part of the sediment was rehomogenized with the supernatant fraction and the sediment, after centrifugation, was called nuclear fraction. The nitrogen and enzyme contents of this fraction varied greatly. Since it was found that sucrose inhibits the $\beta$-glucuronidase activity considerably and to a varying degree, the fractions separated by differential centrifugation were resuspended in distilled water. The mitochondrial fraction for the estimation of succinoxidase, was resuspended in isotonic phosphate buffer of pH 7·4.

Balance sheets, comparing the amount of nitrogen and enzymes in the initial homogenates with that of the sum from the fractions, showed the extent of recovery.

The enzymes were determined by the following methods :

Succinoxidase : manometrically in a Warburg apparatus (Umbreit, Burris and Stauffer, 1951) ; excess cytochrome c, $AlCl_3$ and $CaCl_2$ was added to the reaction mixture. One mm.$^3$ oxygen uptake per hour is designated as one unit.

$\beta$-glucuronidase : $\beta$-glucuronidase activity was determined both in tissue sections and in homogenates. The $\beta$-glucuronidase activity in homogenates was determined after 10 minutes and 1 hour incubation by the method of Talalay, Fishman and Huggins (1946). The addition of saponine or of triton 20 or 100

did not increase the β-glucuronidase activity of homogenates prepared in water. In frozen sections cut 30 μ thick in a cryostat, the β-glucuronidase activity (1 hour incubation) was determined by an adaptation of the above method (Hoch-Ligeti *et al.*, 1964).

Acid phosphatase was determined by the method of Bessey, Lowry and Brock (1946); lactic dehydrogenase by the method of Wroblewski and LaDue (1955); and nitrogen by the Conway method (1958).

### RESULTS

As in the previous experiments (Argus and Hoch-Ligeti, 1961; Hoch-Ligeti *et al.*, 1964), the feeding of DEN did not affect the appetite, growth or general well being of the rats up to a time till tumors as large or larger than the original liver tissue were present. The weight of the liver of DEN treated rats increased over that of the controls before gross or microscopic tumors could be found. The weight of heart, lung, kidney, adrenal and thymus did not differ significantly between the treated and control rats. The chronology of the morphological changes in the liver during tumor development was similar to that described previously (Grundmann and Sieburg, 1962; Hoch-Ligeti *et al.*, 1964). Small disseminated groups of highly atypical cells were observed on the 107th day and small multifocal hepatomas were present on the 120th day. After that time all rats had tumors in the liver. All the tumors were hepatocellular carcinomas and they were generally larger in the females. One female rat, killed on the 191st day of the experiment, had metastases in the lung and an early carcinoma in the kidney.

Because of a possible difference in the response of free and bound β-glucuronidase to the administration of a carcinogen, the effect of feeding DEN was studied both in slices and in homogenates. In Fig. 1 the β-glucuronidase activity per mg. dry tissue in liver slices and homogenates of DEN treated rats is given as per cent of the controls. The increase in the β-glucuronidase concentration in liver slices and homogenates is quite similar.

In Fig. 2 the effects of feeding DEN on the activities of succinoxidase, β-glucuronidase, acid phosphatase and LDH (calculated per mg. nitrogen) are shown as per cent of the control. The time span represented includes values for livers from the treated rats without morphological changes (till 107 days), with preneoplastic changes, and with small and large hepatocellular carcinomas. The succinoxidase concentration was decreased below the control from the 107th day. Similarly to previous findings, the β-glucuronidase activity became elevated around the 70th day. The changes in concentration of these enzymes occurred rather suddenly and, though the degree varied, no definite increment occurred as the tumor development proceeded. In the concentrations of acid phosphatase and LDH of the liver, no significant changes occurred during the whole course of the experiment.

In 3 large tumors the enzyme concentrations were determined. The concentrations of LDH and acid phosphatase did not differ in the tumor and in the surrounding tissue; the mean succinoxidase concentrations were, per 100 mg. wet tissue, in the tumor 212 and in the surrounding tissue 807 units. The mean β-glucuronidase concentrations were 599 and 405 units, respectively.

In Table I are summarized the findings with total homogenates and with subcellular fractions in 17 pairs of rats killed between the 70th and 206th days. The enzyme concentrations are expressed both as units per mg. nitrogen and as percentage of the total found in the fractions. If in several rats the enzyme concentration in a fraction was zero or if the mean of the whole group did not exceed 10% of the total enzyme recovered, the enzyme concentrations in Table I are designated " traces ". The total nitrogen of the homogenate, calculated per

FIG. 1.—β-glucuronidase activity in liver slices and homogenates of DEN treated rats.

▲ = slices.

△ = homogenates.

The ordinate is the ratio of the activity per mg. dry weight for the treated over that for the control rat studied on the same day.

mg. wet tissue, is significantly ($P < 0{\cdot}01$) lower in the treated rats. The mean dry weight of liver was 31·5% for the control and 30·5% for the treated rats. If the nitrogen concentrations are calculated on a dry weight basis, the difference remains significant. The recovery of nitrogen in the fractions of the centrifuged homogenate was between 83 and 108%. The changes in the nitrogen concentration of the fractions are slight, but statistically significant. The nitrogen concentration for the mitochondrial fraction was found to be smaller in the treated than in the control rats in 13/17 instances, in the microsomal fraction in 11/17, and it was higher in the soluble fraction in 16/17 instances.

The enzyme distributions in the subcellular particles follow the same pattern in treated and in control animals. Succinoxidase was found nearly exclusively in the mitochondrial fraction, with a slight spill-over into the microsomal fraction. The concentration of this enzyme in the treated rats is significantly decreased below that in the controls, both in the total homogenate and in the mitochondrial fraction. $\beta$-glucuronidase and acid phosphatase activity is present in every fraction ; the largest portion of $\beta$-glucuronidase is in the mitochondrial, that of

FIG. 2.—Enzyme activities in the liver of DEN treated rats during tumor development.
□ = succinoxidase.
△ = $\beta$-glucuronidase.
● = acid phosphatase.
○ = lactic dehydrogenase.

The ordinate is the ratio of the activity per mg. nitrogen for the treated over that for the control rat studied on the same day.

acid phosphatase in the microsomal fraction. During carcinogenesis the liver $\beta$-glucuronidase concentrations of the total homogenates and of the mitochondrial fraction increased significantly. The concentration of acid phosphatase of the liver homogenate or of its fractions did not differ in the treated and the control rats.

LDH was found only in the microsomal and the soluble fractions, among which it was distributed about equally. In the livers during carcinogenesis, in livers with tumors, or in the tumor tissue itself, an increase of the LDH concentration was not found.

Although the concentration of the succinoxidase in the mitochondrial fraction

TABLE I.—*Mean Nitrogen and Enzyme Concentrations in Total Homogenate and Subcellular Fractions of Livers from Control and DEN Treated Rats*

| | Homogenate | | Fractions of Supernate | | | | | | | |
|---|---|---|---|---|---|---|---|---|---|---|
| | Total | Supernate | Nuclear | | Mitochondrial | | Microsomal | | Soluble | |
| | μg./mg. | μg./mg. | μg./mg. | % | μg./mg. | % | μg./mg. | % | μg./mg. | % |
| Nitrogen : | | | | | | | | | | |
| Control | 27·4† (22·4–32·1) | 18·7 (14·8–22·3) | 2·8 (0·2–6·7) | 11·4 (1·1–19·9) | 2·5 (1·8–3·5) | 12·6 (8·6–18·2) | 5·7 (4·3–8·2) | 29·1 (24·9–33·6) | 9·3 (7·1–11·6) | 45·5 (36·8–53·5) |
| Treated | 24·2* (18·3–29·4) | 16·3* (13·5–19·6) | 2·7 (0·3–6·7) | 10·8 (2·0–21·4) | 2·1* (1·3–2·8) | 10·6 (6·5–15·6) | 5·4 (4·1–7·9) | 27·1 (21·9–31·5) | 9·8* (8·4–12·7) | 49·8 (39·5–60·0) |
| | Units/mg.N | Units/mg.N | Units/mg.N | % | Units/mg.N | % | Units/mg.N | % | Units/mg.N | % |
| Succinoxidase : | | | | | | | | | | |
| Control | 769 (321–1198) | — | Traces | — | 844 (379–1336) | 87·7 (54·7–90·4) | 88 (27–146) | 8·9 (3·2–18·8) | Traces | — |
| Treated | 493* (134–786) | — | Traces | — | 632* (168–792) | 84·5 (44·9–90·8) | 73 (32–181) | 10·8 (2·9–22·4) | Traces | — |
| β-Glucuronidase : | | | | | | | | | | |
| Control | 124 (62–271) | — | 109 (0–202) | 17·5 (0–32·1) | 205 (60–364) | 36·9 (22·0–58·1) | 145 (71–229) | 32·3 (17·1–44·7) | 99 (50–200) | 20·3 (10·1–44·6) |
| Treated | 196* (67–540) | — | 168 (0–354) | 16·6 (0–31·3) | 359* (70–906) | 43·1 (25·7–60·2) | 170 (86–240) | 21·8 (5·6–58·0) | 125 (40–227) | 20·7 (7·9–57·5) |
| Acid phosphatase | | | | | | | | | | |
| Control | 97 (74–180) | 109 (74–182) | 42 (0–128) | 13·0 (2·8–23·8) | 96 (55–204) | 29·7 (17·7–38·3) | 110 (79–199) | 35·6 (18·5–55·9) | 69 (34–120) | 21·6 (13·1–28·7) |
| Treated | 106 (88–158) | 120 (86–204) | 47 (13–120) | 13·4 (3·5–22·8) | 111 (34–255) | 30·9 (20·8–44·5) | 119 (28–224) | 34·3 (25·3–47·3) | 73 (26–107) | 21·4 (10·7–27·0) |
| LDH | | | | | | | | | | |
| Control | — | 2883 (1740–4227) | Traces | — | Traces | — | 2551 (1860–3229) | 46·5 (35·1–56·0) | 3205 (1775–4683 | 53·7 (48·0–64·1) |
| Treated | — | 2831 (1814–4227) | Traces | — | Traces | — | 2790 (1428–4553) | 48·5 (41·4–60·5) | 2982 (1652–4870) | 50·7 (39·2–69·0) |

* Statistically significant $P < 001$.

† Mean (range) for 17 rats.

decreased in the treated rat, the proportion in the sum of all fractions remained the same in treated and in control rats. The same holds true for the increase of $\beta$-glucuronidase, the highest percentage of which occurred in the mitochondrial fraction.

The so-called " relative specific activity ", i.e., the percentage of total enzyme activity over percentage of total nitrogen in a fraction has been calculated (Fig. 3).

FIG. 3. Mean relative specific activity of enzymes in liver of DEN treated and control rats. The means are for 17 rats killed between the 70th and 206th days of experiment. Ordinate: mean relative specific activity of fractions. Abscissa: fractions are represented by their relative nitrogen content, from left to right: nuclear fraction, mitochondrial fraction, microsomal fraction and final soluble fraction. Relative specific activity equals the per cent of total enzyme activity divided by per cent of total protein nitrogen.

The relative specific activity of succinoxidase in treated rats appears to be increased in the mitochondrial fraction, but this result is merely a consequence of the decrease of the percentage of total nitrogen in the fraction. The presence of an enzymatically inactive nitrogen-containing compound, in any one fraction which increases the total nitrogen in the sample, would give a low nitrogen percentage in the subcellular compartments and accordingly high relative specific enzyme activities. If the nitrogen present in a fraction were to derive solely from enzyme protein or if the relative amount of inert nitrogen-containing compounds were to be constant, relative specific activity would be an important concept.

### DISCUSSION

The pattern of the intracellular distribution of enzymes in the liver during carcinogenesis due to feeding of DEN did not differ from that in the liver of untreated rats. The term " precancerous liver " is avoided because actual malignant transformation occurs only in a relatively small number of cells, though morphologic and enzymatic changes can be observed in cells of every part of the liver. The decrease in succinoxidase activity, determined in the homogenate or in the mitochondrial fraction, is in agreement with previous findings during hepatic carcinogenesis caused by different carcinogens, e.g., 1,2 : 5,6-dibenzanthracene (Hoch-Ligeti, 1947), 3′-methyl-4-dimethylaminoazobenzene (Schneider *et al.*, 1953) or 2-acetylaminofluorene (Laird and Miller, 1953). The finding that, although the concentration of succinoxidase was decreased in the mitochondrial fraction, its proportion in the sum of all fractions remained unchanged in rats fed DEN, suggests that a decrease in the number of the morphological units might be a contributing factor to the change in enzyme concentration. A decrease in the number of mitochondria in sections of DEN treated rats was found by direct counting with a computer device (Hoch-Ligeti and Kirsch, unpublished). In the liver tumor itself the succinoxidase activity per unit weight of tissue was found to be about one-fourth that of the surrounding liver. It seems justified to assume that the mitochondria were less numerous than in the surrounding tissue.

With the separation employed, the largest proportion of $\beta$-glucuronidase was associated with the mitochondria, that of acid phosphatase with the microsomes. According to the concept of lysosomes, both enzymes are localized in the same particles, and probably the lysosomes were not satisfactorily separated in the present experiments.

The increase of $\beta$-glucuronidase concentration in the homogenate and in the mitochondrial fraction of liver from DEN treated rats is in agreement with the previous finding, that $\beta$-glucuronidase activity is increased in many cancerous tissues (Fishman and Baker, 1956). The possibility of a change in the ratio of free and intraparticular $\beta$-glucuronidase during carcinogenesis was studied by comparing the enzyme in tissue slices and in tissue homogenates. Although only about a third of the enzyme was available in tissue slices, the rate of increase of enzyme activity in the liver slices and in homogenates of treated rats was about the same, suggesting that the ratio of free to total $\beta$-glucuronidase was not modified by feeding of DEN.

The lack of increase in the concentration of acid phosphatase, presumably localized in the same subcellular particle as $\beta$-glucuronidase, could be explained if a change in the molecular composition of the particle during carcinogenesis is assumed.

During development of hepatic tumors on feeding DEN the total amount and intracellular distribution of LDH remained unchanged.

The recurring problem of all studies of biochemical alteration of tissues during carcinogenesis is whether the changes observed are causally connected with, or concomitant to the development of cancer. Localization and concentration of the same set of enzymes as studied in the present work were investigated in the lung of DEN or DMN treated rats (Hoch-Ligeti, 1966). The changes observed in the succinoxidase and $\beta$-glucuronidase concentrations were the same as in the liver, although they occurred later in the experiment. Since the frequency of tumor development differs greatly in the two organs, it is suggested that the enzymatic changes reflect systemic alterations preparatory and concomitant to cancer development but that they may not be the necessary final metabolic step to neoplastic transformation of cells.

SUMMARY

1. In rats fed DEN the concentrations and subcellular distributions of succinoxidase, $\beta$-glucuronidase, acid phosphatase and lactic dehydrogenase were studied. These enzymes were chosen because of their different intracellular distribution.

2. Expressed per mg. nitrogen, the concentration of succinoxidase decreased in the homogenate and in the mitochondrial fraction, that of $\beta$-glucuronidase increased, the concentrations of acid phosphatase and lactic dehydrogenase remained unchanged in precancerous liver, in tumor-free areas of livers with tumor and in hepatic tumors.

3. The localization and proportion of the enzymes in the subcellular compartments did not differ in the livers of treated and control rats.

4. The changes in the enzyme concentrations appear to be a consequence of changes in the number and in structure of the morphological units in which the enzyme is localized.

This work was supported by Grant P–2661 from the Charles McCamic Memorial Grant for Cancer Research from The American Cancer Society.

REFERENCES

ALLFREY, V.—(1959) 'The Cell', edited by J. Brachet and A. E. Mirsky. New York and London (Academic Press), Vol. 1, pp. 193–290.

APPELMANS, F., WATTIAUX, R. AND DE DUVE, C.—(1955) *Biochem. J.*, **59**, 438.

ARGUS, M. F. AND HOCH-LIGETI, C.—(1961) *J. natn. Cancer Inst.*, **27**, 695.

BESSEY, O. A., LOWRY, O. H. AND BROCK, M. J.—(1946) *J. biol. Chem.*, **164**, 321.

BRUNNGRABER, E. G. AND ABOOD, L. G.—(1960) *J. biol. Chem.*, **235**, 1847.

CONWAY, E. J.—(1958) 'Microdiffusion and Volumetric Error'. New York (MacMillan).

DE DUVE, C.—(1960) *Bull. Soc. Chim. biol.*, **42**, 11.

DE DUVE, C., PRESSMAN, B. C., GIANETTO, R., WATTIAUX, R. AND APPELMANS, F.—(1955) *Biochem. J.*, **60**, 604.

FISHMAN, W. H. AND BAKER, J. R.—(1956) *J. Histochem. Cytochem.*, **4**, 570.

GREEN, D. E. AND HATEFI, Y.—(1961) *Science*, **133**, 13.

GRUNDMANN, E. AND SIEBURG, H.—(1962) *Beitr. path. Anat.*, **126**, 57.

HESS, R., SCARPELLI, D. G. AND PEARSE, A. G.—(1958) *Nature, Lond.*, **181**, 1531.

HOCH-LIGETI, C.—(1947) *Cancer Res.*, **7**, 148.

HOCH-LIGETI, C., LOBL, L. T. AND ARVIN, J. M.—(1964) *Br. J. Cancer*, **18**, 271.

HOCH-LIGETI, C.—(1966, in press) in 'Lung Tumors in Animals'. Proceedings of the Conference at Perugia, edited by L. Severi, Division of Cancer Research, Perugia, Italy.

HOU, C. T. AND REES, K. R.—(1961) *Br. J. Cancer*, **15**, 624.

LAIRD, A. K. AND MILLER, E. C.—(1953) *Cancer Res.*, **13**, 464.

NACHLAS, M. M., WALKER, D. G. AND SELIGMAN, A. M.—(1958) *J. biophys. biochem. Cytol.*, **4**, 29.

NOVIKOFF, A. B.—(1960) 'Cell Physiology of Neoplasia'. Austin, Texas (The University of Texas Press), p. 219.

NOVIKOFF, A. B.—(1961) 'The Cell', edited by J. Brachet and A. E. Mirsky. New York and London (Academic Press), Vol. 2, pp. 344–345.

SCHNEIDER, W. C.—(1946) *Cancer Res.*, **6**, 685.

SCHNEIDER, W. C.—(1959) *Adv. Enzymol.*, **21**, 1.

Schneider, W. C. and Hogeboom, G. H.—(1950) *J. biol. Chem.*, **183**, 123.
Schneider, W. C., Hogeboom, G. H., Shelton, E. and Striebich, M. J.—(1953) *Cancer Res.*, **13**, 285.
Talalay, P., Fishman, W. H. and Huggins, C.—(1946) *J. biol. Chem.*, **166**, 757.
Umbreit, W. W., Burris, R. H. and Stauffer, J. F.—(1951) ' Manometric Techniques and Tissue Metabolism '. Minneapolis, Minnesota (Burgess Publishing Co.).
Vesell, E. S. and Bearn, A. G.—(1962) *Proc. Soc. exp. Biol. Med.*, **111**, 100.
Walker, P. G.—(1952) *Biochem. J.*, **51**, 223.
Wroblewski, F. and LaDue, J. S.—(1955) *Proc. Soc. exp. Biol. Med.*, **90**, 210.